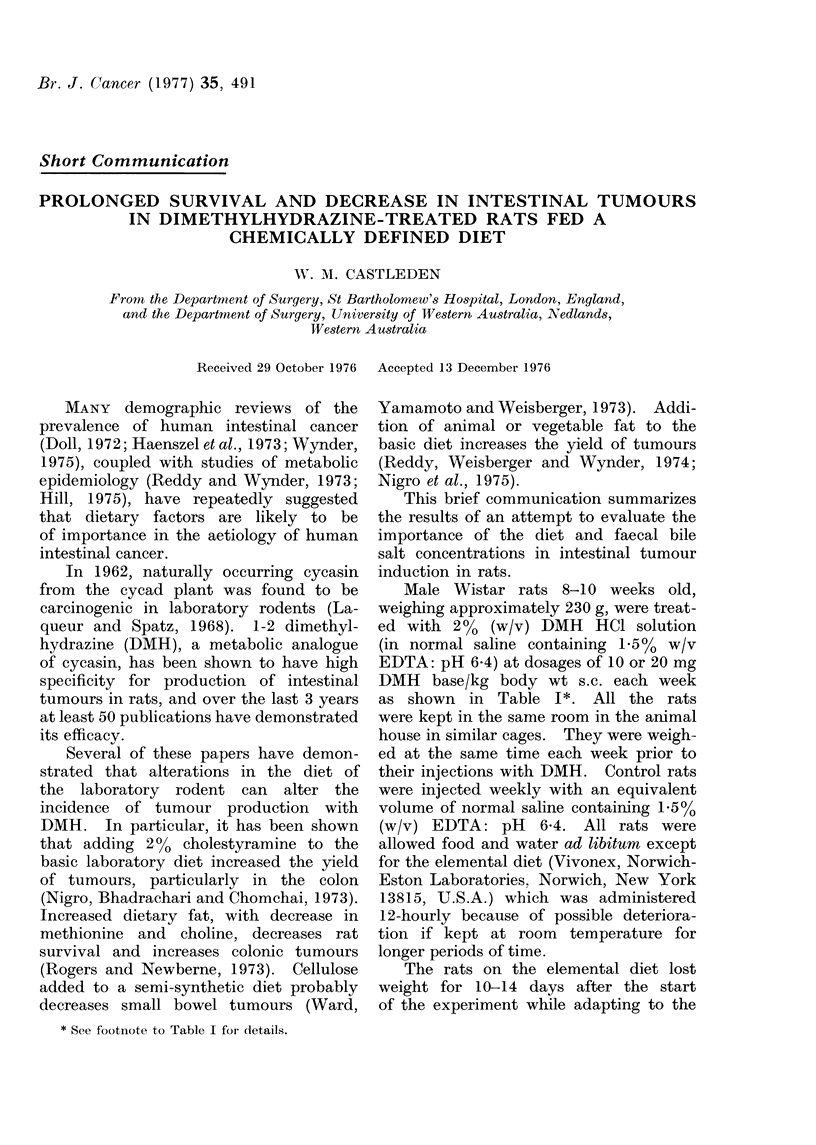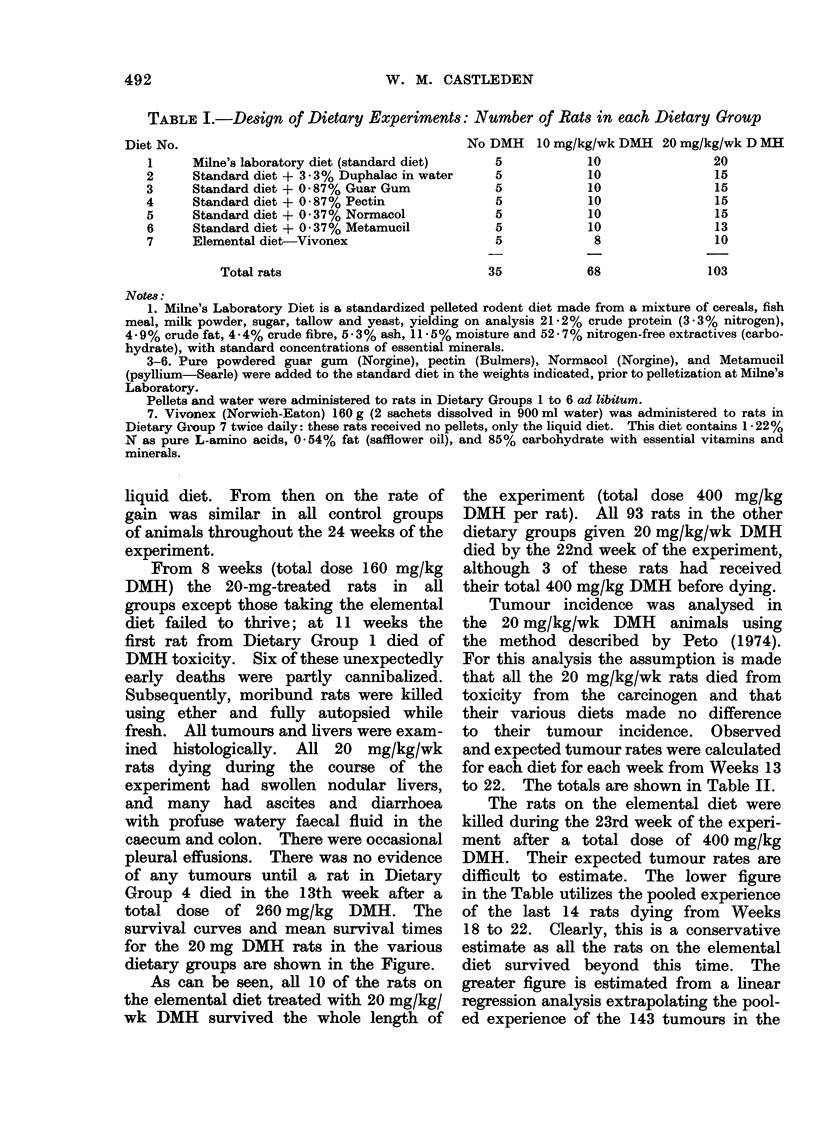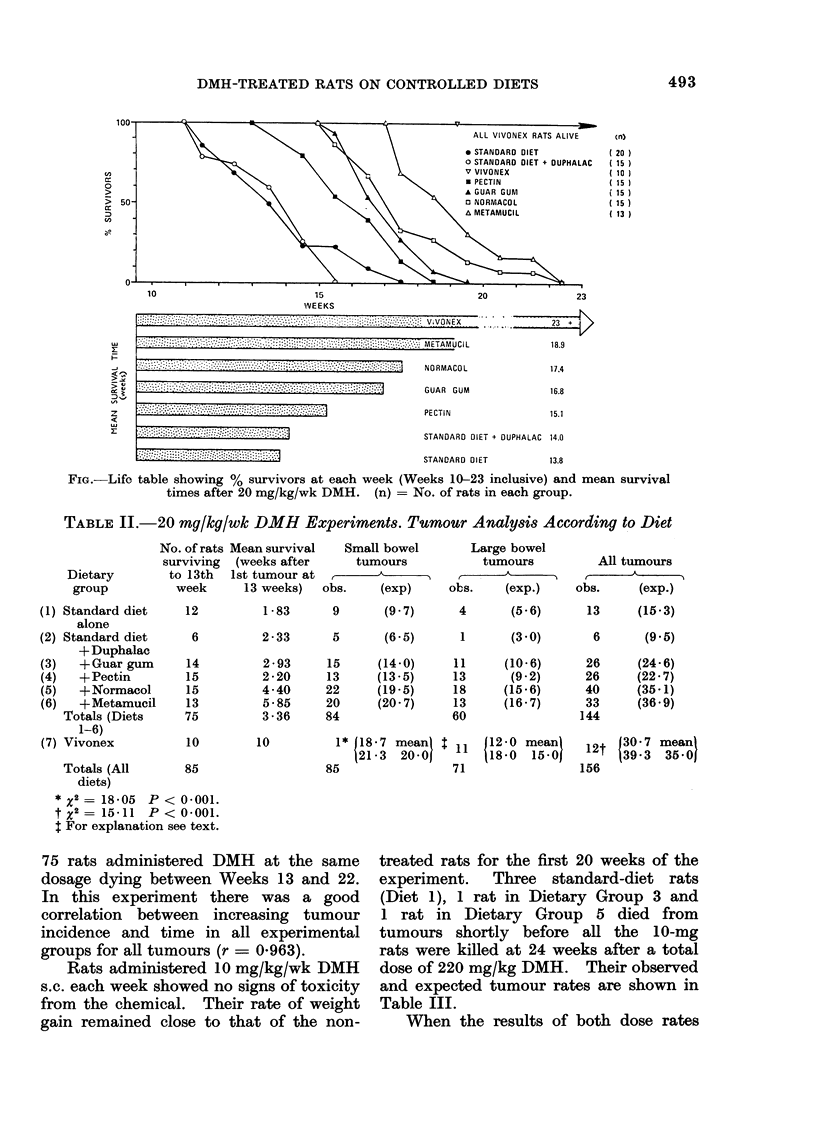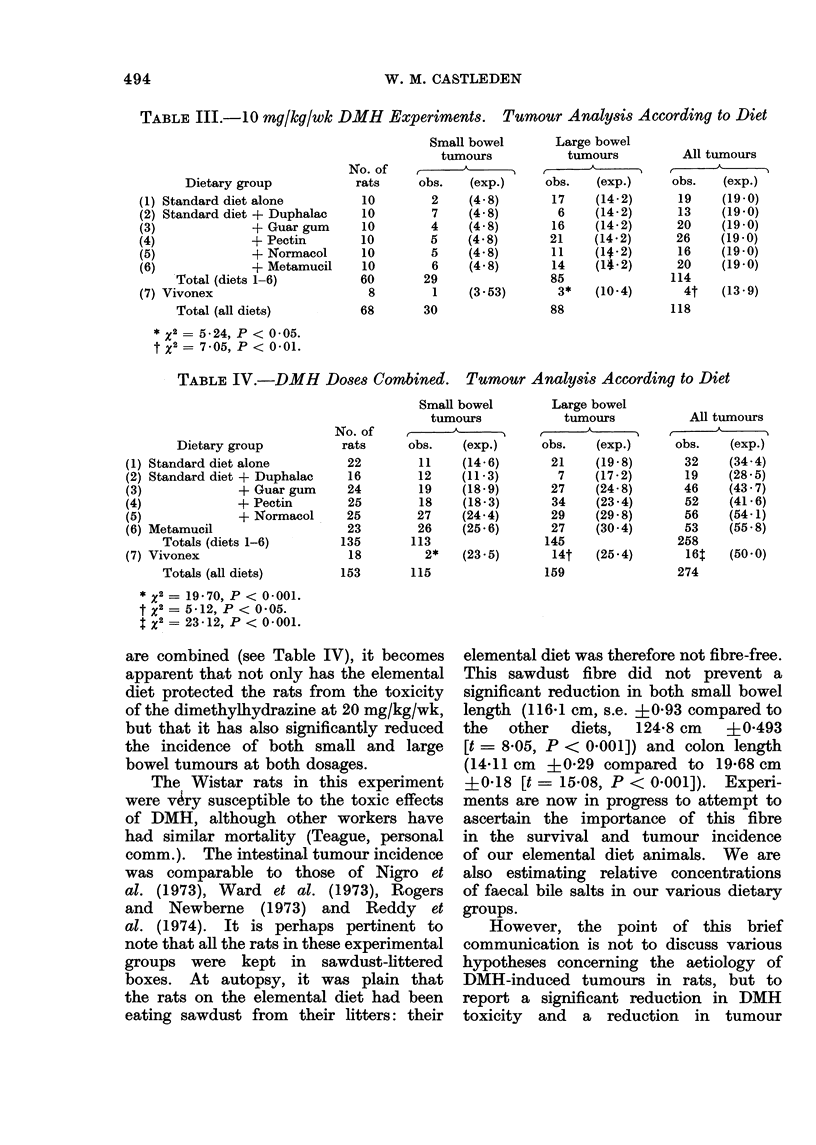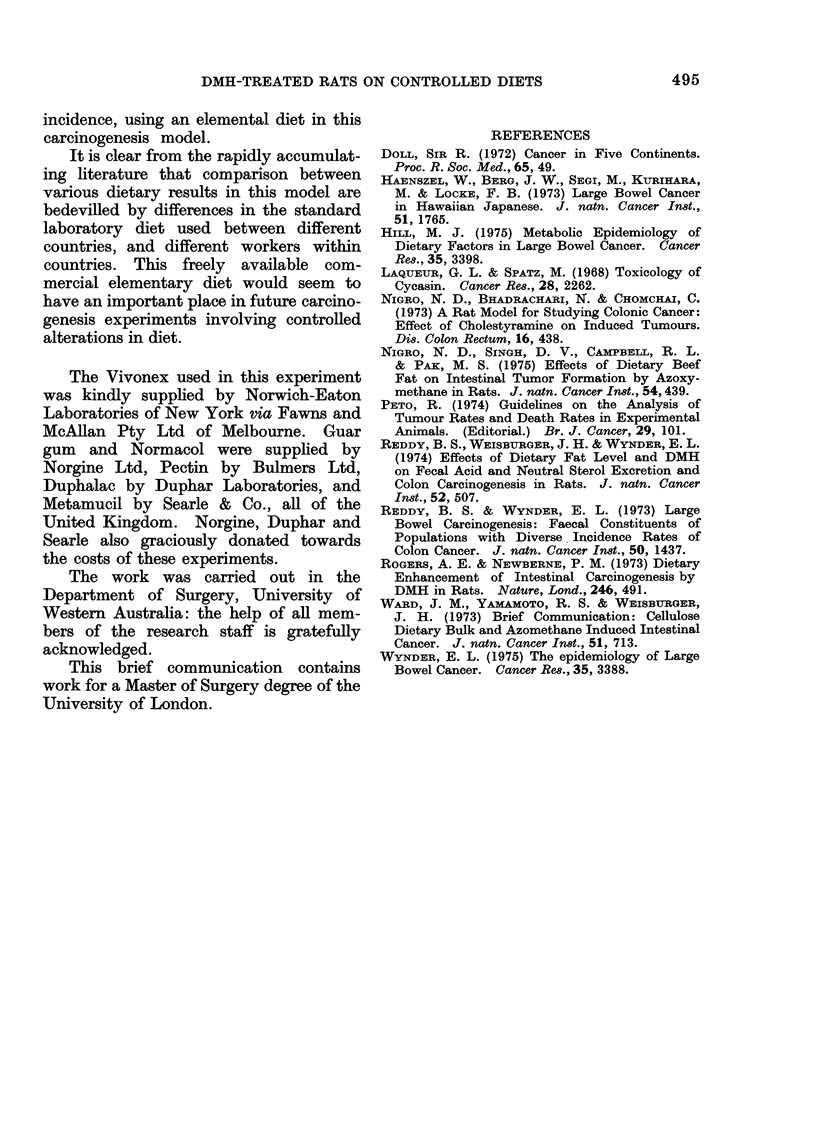# Prolonged survival and decrease in intestinal tumours in dimethylhydrazine-treated rats fed a chemically defined diet.

**DOI:** 10.1038/bjc.1977.74

**Published:** 1977-04

**Authors:** W. M. Castleden


*Br. J. Cancer* (1977) **35**, 491

***Short Communication***

### PROLONGED SURVIVAL AND DECREASE IN INTESTINAL TUMOURS IN DIMETHYLHYDRAZINE-TREATED RATS FED A CHEMICALLY DEFINED DIET

W. M. CASTLEDEN

*From the Department of Surgery, St Bartholomew's Hospital, London, England, and the Department of Surgery, University of Western Australia, Nedlands, Western Australia*

Received 29 October 1976 Accepted 13 December 1976

MANY demographic reviews of the prevalence of human intestinal cancer (Doll, 1972; Haenszel *et al.*, 1973; Wynder, 1975), coupled with studies of metabolic epidemiology (Reddy and Wynder, 1973; Hill, 1975), have repeatedly suggested that dietary factors are likely to be of importance in the aetiology of human intestinal cancer.

In 1962, naturally occurring cycasin from the cycad plant was found to be carcinogenic in laboratory rodents (Laqueur and Spatz, 1968). 1-2 dimethylhydrazine (DMH), a metabolic analogue of cycasin, has been shown to have high specificity for production of intestinal tumours in rats, and over the last 3 years at least 50 publications have demonstrated its efficacy.

Several of these papers have demonstrated that alterations in the diet of the laboratory rodent can alter the incidence of tumour production with DMH. In particular, it has been shown that adding 2% cholestyramine to the basic laboratory diet increased the yield of tumours, particularly in the colon (Nigro, Bhadrachari and Chomchai, 1973). Increased dietary fat, with decrease in methionine and choline, decreases rat survival and increases colonic tumours (Rogers and Newberne, 1973). Cellulose added to a semi-synthetic diet probably decreases small bowel tumours (Ward, Yamamoto and Weisberger, 1973). Addition of animal or vegetable fat to the basic diet increases the yield of tumours (Reddy, Weisberger and Wynder, 1974; Nigro *et al.*, 1975).

This brief communication summarizes the results of an attempt to evaluate the importance of the diet and faecal bile salt concentrations in intestinal tumour induction in rats.

Male Wistar rats 8–10 weeks old, weighing approximately 230 g, were treated with 2% (w/v) DMH HCl solution (in normal saline containing 1·5% w/v EDTA: pH 6·4) at dosages of 10 or 20 mg DMH base/kg body wt s.c. each week as shown in Table I*. All the rats were kept in the same room in the animal house in similar cages. They were weighed at the same time each week prior to their injections with DMH. Control rats were injected weekly with an equivalent volume of normal saline containing 1·5% (w/v) EDTA: pH 6·4. All rats were allowed food and water *ad libitum* except for the elemental diet (Vivonex, Norwich-Eston Laboratories, Norwich, New York 13815, U.S.A.) which was administered 12-hourly because of possible deterioration if kept at room temperature for longer periods of time.

The rats on the elemental diet lost weight for 10–14 days after the start of the experiment while adapting to the

* See footnote to Table I for details.

TABLE I.—*Design of Dietary Experiments: Number of Rats in each Dietary Group*

| Diet No. | | No DMH | 10 mg/kg/wk DMH | 20 mg/kg/wk D MH |
|---|---|---|---|---|
| 1 | Milne's laboratory diet (standard diet) | 5 | 10 | 20 |
| 2 | Standard diet + 3·3% Duphalac in water | 5 | 10 | 15 |
| 3 | Standard diet + 0·87% Guar Gum | 5 | 10 | 15 |
| 4 | Standard diet + 0·87% Pectin | 5 | 10 | 15 |
| 5 | Standard diet + 0·37% Normacol | 5 | 10 | 15 |
| 6 | Standard diet + 0·37% Metamucil | 5 | 10 | 13 |
| 7 | Elemental diet—Vivonex | 5 | 8 | 10 |
| | Total rats | 35 | 68 | 103 |

*Notes:*

1. Milne's Laboratory Diet is a standardized pelleted rodent diet made from a mixture of cereals, fish meal, milk powder, sugar, tallow and yeast, yielding on analysis 21·2% crude protein (3·3% nitrogen), 4·9% crude fat, 4·4% crude fibre, 5·3% ash, 11·5% moisture and 52·7% nitrogen-free extractives (carbohydrate), with standard concentrations of essential minerals.

3–6. Pure powdered guar gum (Norgine), pectin (Bulmers), Normacol (Norgine), and Metamucil (psyllium—Searle) were added to the standard diet in the weights indicated, prior to pelletization at Milne's Laboratory.

Pellets and water were administered to rats in Dietary Groups 1 to 6 *ad libitum*.

7. Vivonex (Norwich-Eaton) 160 g (2 sachets dissolved in 900 ml water) was administered to rats in Dietary Group 7 twice daily: these rats received no pellets, only the liquid diet. This diet contains 1·22% N as pure L-amino acids, 0·54% fat (safflower oil), and 85% carbohydrate with essential vitamins and minerals.

liquid diet. From then on the rate of gain was similar in all control groups of animals throughout the 24 weeks of the experiment.

From 8 weeks (total dose 160 mg/kg DMH) the 20-mg-treated rats in all groups except those taking the elemental diet failed to thrive; at 11 weeks the first rat from Dietary Group 1 died of DMH toxicity. Six of these unexpectedly early deaths were partly cannibalized. Subsequently, moribund rats were killed using ether and fully autopsied while fresh. All tumours and livers were examined histologically. All 20 mg/kg/wk rats dying during the course of the experiment had swollen nodular livers, and many had ascites and diarrhoea with profuse watery faecal fluid in the caecum and colon. There were occasional pleural effusions. There was no evidence of any tumours until a rat in Dietary Group 4 died in the 13th week after a total dose of 260 mg/kg DMH. The survival curves and mean survival times for the 20 mg DMH rats in the various dietary groups are shown in the Figure.

As can be seen, all 10 of the rats on the elemental diet treated with 20 mg/kg/wk DMH survived the whole length of the experiment (total dose 400 mg/kg DMH per rat). All 93 rats in the other dietary groups given 20 mg/kg/wk DMH died by the 22nd week of the experiment, although 3 of these rats had received their total 400 mg/kg DMH before dying.

Tumour incidence was analysed in the 20 mg/kg/wk DMH animals using the method described by Peto (1974). For this analysis the assumption is made that all the 20 mg/kg/wk rats died from toxicity from the carcinogen and that their various diets made no difference to their tumour incidence. Observed and expected tumour rates were calculated for each diet for each week from Weeks 13 to 22. The totals are shown in Table II.

The rats on the elemental diet were killed during the 23rd week of the experiment after a total dose of 400 mg/kg DMH. Their expected tumour rates are difficult to estimate. The lower figure in the Table utilizes the pooled experience of the last 14 rats dying from Weeks 18 to 22. Clearly, this is a conservative estimate as all the rats on the elemental diet survived beyond this time. The greater figure is estimated from a linear regression analysis extrapolating the pooled experience of the 143 tumours in the

FIG.—Life table showing % survivors at each week (Weeks 10–23 inclusive) and mean survival times after 20 mg/kg/wk DMH. (n) = No. of rats in each group.

TABLE II.—20 *mg/kg/wk DMH Experiments. Tumour Analysis According to Diet*

| Dietary group | No. of rats surviving to 13th week | Mean survival (weeks after 1st tumour at 13 weeks) | Small bowel tumours obs. | Small bowel tumours (exp) | Large bowel tumours obs. | Large bowel tumours (exp.) | All tumours obs. | All tumours (exp.) |
|---|---|---|---|---|---|---|---|---|
| (1) Standard diet alone | 12 | 1·83 | 9 | (9·7) | 4 | (5·6) | 13 | (15·3) |
| (2) Standard diet +Duphalac | 6 | 2·33 | 5 | (6·5) | 1 | (3·0) | 6 | (9·5) |
| (3) +Guar gum | 14 | 2·93 | 15 | (14·0) | 11 | (10·6) | 26 | (24·6) |
| (4) +Pectin | 15 | 2·20 | 13 | (13·5) | 13 | (9·2) | 26 | (22·7) |
| (5) +Normacol | 15 | 4·40 | 22 | (19·5) | 18 | (15·6) | 40 | (35·1) |
| (6) +Metamucil | 13 | 5·85 | 20 | (20·7) | 13 | (16·7) | 33 | (36·9) |
| Totals (Diets 1–6) | 75 | 3·36 | 84 | | 60 | | 144 | |
| (7) Vivonex | 10 | 10 | 1* | {18·7 mean<br>21·3 20·0} ‡ | 11 | {12·0 mean<br>18·0 15·0} | 12† | {30·7 mean<br>39·3 35·0} |
| Totals (All diets) | 85 | | 85 | | 71 | | 156 | |

* $\chi^2 = 18{\cdot}05$ $P < 0{\cdot}001$.

† $\chi^2 = 15{\cdot}11$ $P < 0{\cdot}001$.

‡ For explanation see text.

75 rats administered DMH at the same dosage dying between Weeks 13 and 22. In this experiment there was a good correlation between increasing tumour incidence and time in all experimental groups for all tumours ($r = 0{\cdot}963$).

Rats administered 10 mg/kg/wk DMH s.c. each week showed no signs of toxicity from the chemical. Their rate of weight gain remained close to that of the non-treated rats for the first 20 weeks of the experiment. Three standard-diet rats (Diet 1), 1 rat in Dietary Group 3 and 1 rat in Dietary Group 5 died from tumours shortly before all the 10-mg rats were killed at 24 weeks after a total dose of 220 mg/kg DMH. Their observed and expected tumour rates are shown in Table III.

When the results of both dose rates

TABLE III.—10 *mg/kg/wk DMH Experiments. Tumour Analysis According to Diet*

| Dietary group | No. of rats | Small bowel tumours obs. | Small bowel tumours (exp.) | Large bowel tumours obs. | Large bowel tumours (exp.) | All tumours obs. | All tumours (exp.) |
|---|---|---|---|---|---|---|---|
| (1) Standard diet alone | 10 | 2 | (4·8) | 17 | (14·2) | 19 | (19·0) |
| (2) Standard diet + Duphalac | 10 | 7 | (4·8) | 6 | (14·2) | 13 | (19·0) |
| (3) + Guar gum | 10 | 4 | (4·8) | 16 | (14·2) | 20 | (19·0) |
| (4) + Pectin | 10 | 5 | (4·8) | 21 | (14·2) | 26 | (19·0) |
| (5) + Normacol | 10 | 5 | (4·8) | 11 | (14·2) | 16 | (19·0) |
| (6) + Metamucil | 10 | 6 | (4·8) | 14 | (14·2) | 20 | (19·0) |
| Total (diets 1–6) | 60 | 29 | | 85 | | 114 | |
| (7) Vivonex | 8 | 1 | (3·53) | 3* | (10·4) | 4† | (13·9) |
| Total (all diets) | 68 | 30 | | 88 | | 118 | |

* $\chi^2 = 5 \cdot 24$, $P < 0 \cdot 05$.

† $\chi^2 = 7 \cdot 05$, $P < 0 \cdot 01$.

TABLE IV.—*DMH Doses Combined. Tumour Analysis According to Diet*

| Dietary group | No. of rats | Small bowel tumours obs. | Small bowel tumours (exp.) | Large bowel tumours obs. | Large bowel tumours (exp.) | All tumours obs. | All tumours (exp.) |
|---|---|---|---|---|---|---|---|
| (1) Standard diet alone | 22 | 11 | (14·6) | 21 | (19·8) | 32 | (34·4) |
| (2) Standard diet + Duphalac | 16 | 12 | (11·3) | 7 | (17·2) | 19 | (28·5) |
| (3) + Guar gum | 24 | 19 | (18·9) | 27 | (24·8) | 46 | (43·7) |
| (4) + Pectin | 25 | 18 | (18·3) | 34 | (23·4) | 52 | (41·6) |
| (5) + Normacol | 25 | 27 | (24·4) | 29 | (29·8) | 56 | (54·1) |
| (6) Metamucil | 23 | 26 | (25·6) | 27 | (30·4) | 53 | (55·8) |
| Totals (diets 1–6) | 135 | 113 | | 145 | | 258 | |
| (7) Vivonex | 18 | 2* | (23·5) | 14† | (25·4) | 16‡ | (50·0) |
| Totals (all diets) | 153 | 115 | | 159 | | 274 | |

* $\chi^2 = 19 \cdot 70$, $P < 0 \cdot 001$.

† $\chi^2 = 5 \cdot 12$, $P < 0 \cdot 05$.

‡ $\chi^2 = 23 \cdot 12$, $P < 0 \cdot 001$.

are combined (see Table IV), it becomes apparent that not only has the elemental diet protected the rats from the toxicity of the dimethylhydrazine at 20 mg/kg/wk, but that it has also significantly reduced the incidence of both small and large bowel tumours at both dosages.

The Wistar rats in this experiment were very susceptible to the toxic effects of DMH, although other workers have had similar mortality (Teague, personal comm.). The intestinal tumour incidence was comparable to those of Nigro *et al.* (1973), Ward *et al.* (1973), Rogers and Newberne (1973) and Reddy *et al.* (1974). It is perhaps pertinent to note that all the rats in these experimental groups were kept in sawdust-littered boxes. At autopsy, it was plain that the rats on the elemental diet had been eating sawdust from their litters: their elemental diet was therefore not fibre-free. This sawdust fibre did not prevent a significant reduction in both small bowel length (116·1 cm, s.e. $\pm 0 \cdot 93$ compared to the other diets, 124·8 cm $\pm 0 \cdot 493$ [$t = 8 \cdot 05$, $P < 0 \cdot 001$]) and colon length (14·11 cm $\pm 0 \cdot 29$ compared to 19·68 cm $\pm 0 \cdot 18$ [$t = 15 \cdot 08$, $P < 0 \cdot 001$]). Experiments are now in progress to attempt to ascertain the importance of this fibre in the survival and tumour incidence of our elemental diet animals. We are also estimating relative concentrations of faecal bile salts in our various dietary groups.

However, the point of this brief communication is not to discuss various hypotheses concerning the aetiology of DMH-induced tumours in rats, but to report a significant reduction in DMH toxicity and a reduction in tumour incidence, using an elemental diet in this carcinogenesis model.

It is clear from the rapidly accumulating literature that comparison between various dietary results in this model are bedevilled by differences in the standard laboratory diet used between different countries, and different workers within countries. This freely available commercial elementary diet would seem to have an important place in future carcinogenesis experiments involving controlled alterations in diet.

The Vivonex used in this experiment was kindly supplied by Norwich-Eaton Laboratories of New York *via* Fawns and McAllan Pty Ltd of Melbourne. Guar gum and Normacol were supplied by Norgine Ltd, Pectin by Bulmers Ltd, Duphalac by Duphar Laboratories, and Metamucil by Searle & Co., all of the United Kingdom. Norgine, Duphar and Searle also graciously donated towards the costs of these experiments.

The work was carried out in the Department of Surgery, University of Western Australia: the help of all members of the research staff is gratefully acknowledged.

This brief communication contains work for a Master of Surgery degree of the University of London.

### REFERENCES

Doll, Sir R. (1972) Cancer in Five Continents. *Proc. R. Soc. Med.*, **65**, 49.

Haenszel, W., Berg, J. W., Segi, M., Kurihara, M. & Locke, F. B. (1973) Large Bowel Cancer in Hawaiian Japanese. *J. natn. Cancer Inst.*, **51**, 1765.

Hill, M. J. (1975) Metabolic Epidemiology of Dietary Factors in Large Bowel Cancer. *Cancer Res.*, **35**, 3398.

Laqueur, G. L. & Spatz, M. (1968) Toxicology of Cycasin. *Cancer Res.*, **28**, 2262.

Nigro, N. D., Bhadrachari, N. & Chomchai, C. (1973) A Rat Model for Studying Colonic Cancer: Effect of Cholestyramine on Induced Tumours. *Dis. Colon Rectum*, **16**, 438.

Nigro, N. D., Singh, D. V., Campbell, R. L. & Pak, M. S. (1975) Effects of Dietary Beef Fat on Intestinal Tumor Formation by Azoxymethane in Rats. *J. natn. Cancer Inst.*, **54**, 439.

Peto, R. (1974) Guidelines on the Analysis of Tumour Rates and Death Rates in Experimental Animals. (Editorial.) *Br. J. Cancer*, **29**, 101.

Reddy, B. S., Weisburger, J. H. & Wynder, E. L. (1974) Effects of Dietary Fat Level and DMH on Fecal Acid and Neutral Sterol Excretion and Colon Carcinogenesis in Rats. *J. natn. Cancer Inst.*, **52**, 507.

Reddy, B. S. & Wynder, E. L. (1973) Large Bowel Carcinogenesis: Faecal Constituents of Populations with Diverse Incidence Rates of Colon Cancer. *J. natn. Cancer Inst.*, **50**, 1437.

Rogers, A. E. & Newberne, P. M. (1973) Dietary Enhancement of Intestinal Carcinogenesis by DMH in Rats. *Nature, Lond.*, **246**, 491.

Ward, J. M., Yamamoto, R. S. & Weisburger, J. H. (1973) Brief Communication: Cellulose Dietary Bulk and Azomethane Induced Intestinal Cancer. *J. natn. Cancer Inst.*, **51**, 713.

Wynder, E. L. (1975) The epidemiology of Large Bowel Cancer. *Cancer Res.*, **35**, 3388.